# Prediction of Tensile Strength of 3D Printed Bronze PLA Part Using Response Surface Modelling

**DOI:** 10.12688/f1000research.70641.1

**Published:** 2021-10-11

**Authors:** Chockalingam Palanisamy, Sugendran Nagarajan

**Affiliations:** 1 Faculty of Engineering and Technology, Multimedia University, Melaka, Melaka, 75450, Malaysia

**Keywords:** 3D Printing, RSM, Bronze PLA, Tensile Strength

## Abstract

**Background **- 3D printing is a dynamic process with many process parameters influencing the product, including the type of the material; it is often difficult to understand the combined influence of these parameters.

**Purpose** - The tensile strength of 3D printed parts is important for the functionality of components. The effects of process parameters on tensile strength must therefore be examined. The objective of this study is to develop a response surface model (RSM) to predict the final quality of a 3D printed bronze part from a different set of input parameters.

**Methods** - The tensile test specimen was built in a Makerbot 3D printer with bronze polylactic acid (PLA) material. The three controllable input parameters were; thickness of layers, number of shells, and infill density. The three levels of layer thickness were 0.1mm, 0.2mm and 0.3mm. The number of shells was 2, 3 and 4. The infill densities were 20%, 30% and 40%. A tensile experiment tested the strength of the specimens. RSM is a statistical approach for modelling and analyzing how different variables affect the response of interest, and for optimizing it.

**Results - **The result obtained shows that the specimen with a high layer thickness of 0.3mm and infill density of 40% is the best among all the other parameters. Finally, the regression equation produced was used for random values of layer thickness, the number of shells, and infill density, to see whether the values obtained from the tests fall into the range of experimental data.

**Conclusion** - Infill density and layer thickness are the two criteria that significantly influence the tensile property. The number of shells has the least influence on the tensile property. However, the best tensile strength is the part printed with higher infill density, a greater number of shells, and higher layer thickness.

## Introduction

Competitiveness in the global marketplace is increasing and manufacturers must seek ways to increase production output and quality and reduce the costs of production. 3D printing, a very recent innovation and state-of-the-art technology, is a solution that could meet their requirements. ^1^ Bronze - polylactic acid (PLA) is the most widely used material in the 3D printing of most mechanical parts. 3D printing produces quality and defect-free objects. However, 3D printed parts do not have high tensile properties. With a combination of input parameters, parts can be printed with enhanced tensile properties. A large number of parameters influence the properties of the product; it is often difficult to understand the combination of these parameters. ^2^ Among other considerations, printing parameters such as the thickness of each layer, number of shells and density of the infill have a major impact on the quality and performance of 3D printed components. Since tensile properties are important for the functioning of components, the effect of process parameters on mechanical properties will be studied. In this research, bronze material is used because it has not been thoroughly studied, unlike ABS or PLA. Finally, a response surface model (RSM) using Minitab 18 ( Minitab, RRID:SCR_014483) software (alternatively, R Software) is developed.

## Methods

**Figure 1.**
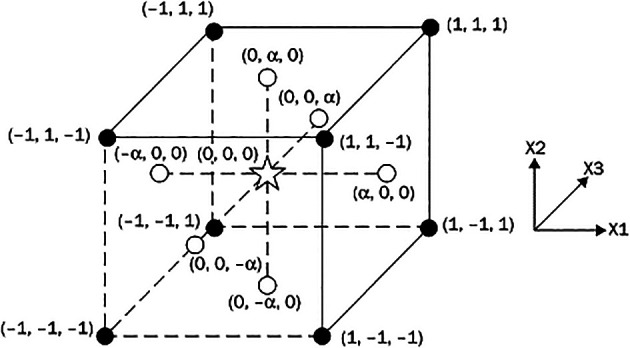
Central composite design.

The three variable input parameters are layer thickness, number of shells, and infill density. In this study, the levels of the input parameters are: layer thickness (0.1 mm, 0.2 mm and 0.3 mm); infill percentage density (10%, 20%, and 40%); number of shells (2, 3, and 4)—all with a constant print speed of 50 mm/s. For the design of the experiment (DOE), central composite design (CCD) ( Figure 1) with three input parameters ( Table 1)—layer thickness, number of shells, and infill density with three levels—was used in this investigation. ^3^ Based on the CCD Design Matrix ( Table 2), 15 sets of samples were 3D printed using Makerbot Replicator with bronze PLA material.

**Table 1.** Input parameters and their levels.

Parameter	Levels		
	−1	0	1
Layer thickness	0.1	0.2	0.3
Infill density	20	30	40
Number of shells	2	3	4

**Table 2.** Experiment plan and tensile test results.

No.	Input parameters			Tensile test results, kN
	Layer thickness	No. of shells	Infill density	
1	0.1	2	20	0.287
2	0.3	2	20	0.582
3	0.1	4	20	0.905
4	0.3	4	20	0.669
5	0.1	2	40	0.549
6	0.3	2	40	0.914
7	0.1	4	40	0.657
8	0.3	4	40	0.900
9	0.1	3	30	0.505
10	0.3	3	30	0.633
11	0.2	2	30	0.864
12	0.2	4	30	0.605
13	0.2	3	20	0.708
14	0.2	3	40	0.71
15	0.2	3	30	0.66

Tensile testing was carried out on the samples and data was recorded. ^4^ Using this data, a response surface model was created using Minitab 18 software (alternatively R Software). An Analysis of Variance (ANOVA) was also performed, with the independent variables, layer thickness, number of shells and infill density, to find out the factor settings that optimize the dependent variable tensile strength. Finally, a Pareto chart of the standardized effects was created to compare the relative magnitude and the statistical significance of main, square, and interaction effects of independent variables which contribute to the most variability to the dependent variable tensile strength, which also plots a reference line to indicate which effects are statistically significant.

## Results

Tensile testing was conducted on specimens that were printed according to the ASTM D256 specification, using the InstraonTensile tester, Instron 3360 Series dual-column table-frame equipment; the data was collected using Instron Bluehill 3 software. The results obtained from the tensile test are shown in Table 2. The results from the testing were then fed into Minitab Software to develop the response surface model of the Bronze-PLA specimen. The regression equation was obtained from the software to compute the theoretical data. Instron’s 3360 Series Dual Column Table Frames were used to conduct the tensile test. This equipment is capable of running both tensile and compression tests.

## Discussion

**Figure 2.**
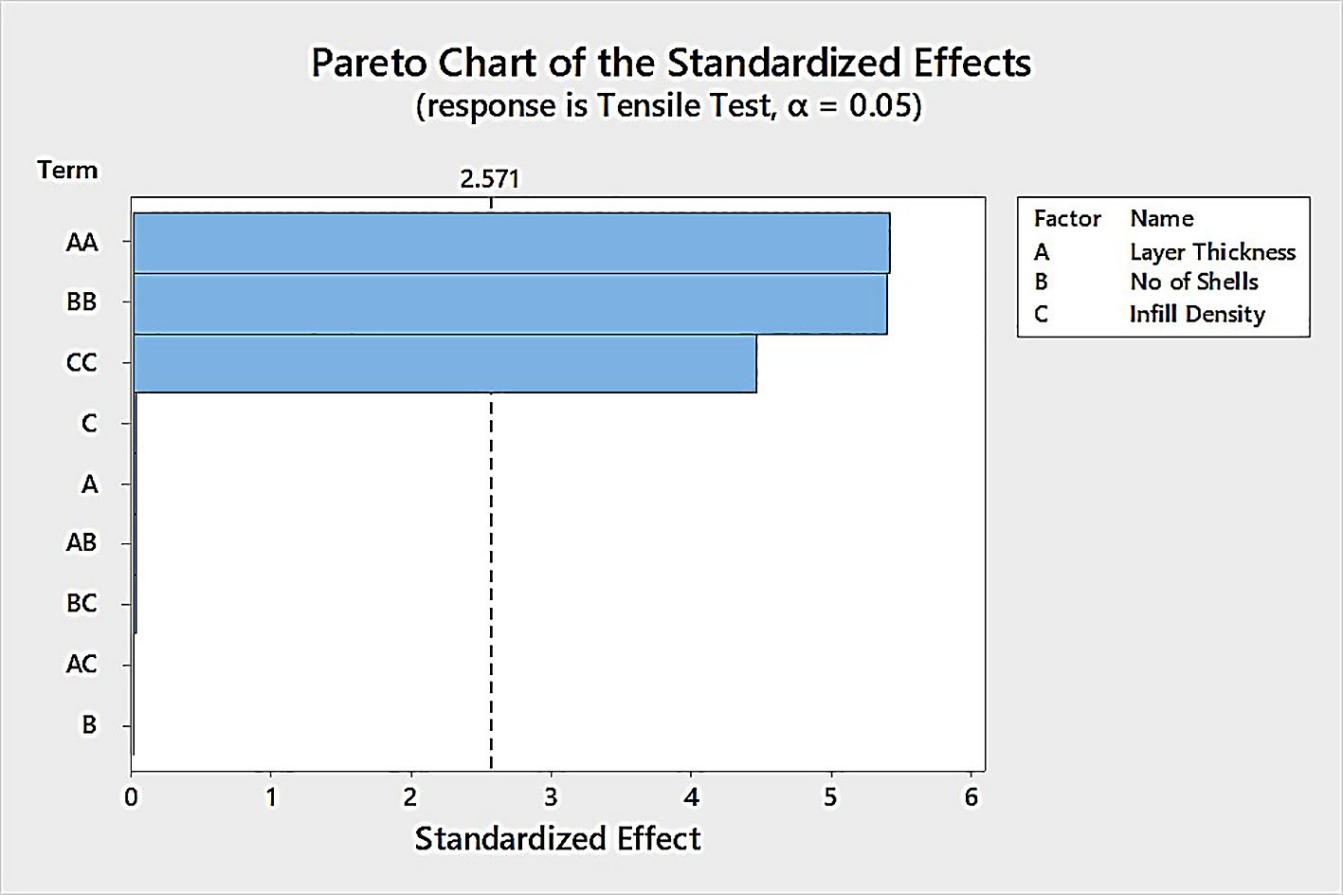
Pareto chart for tensile strength.

Based on the ANOVA analysis ( Table 3), the Pareto chart ( Figure 2) indicates that the factor square terms of the layer thickness, number of shells, and infill density are significant factors. ^5^

**Table 3.** ANOVA coded values.

Term	Coef	SE Coef	T-Value	p-Value	VIF
Constant	0.664	0.081	8.20	0.00	
Layer thickness	0.079	0.047	1.67	0.15	1.0
No. of shells	0.054	0.047	1.13	0.30	1.0
Infill density	0.058	0.047	1.22	0.27	1.0
Layer thickness ^2^	−0.096	0.094	−1.02	0.35	1.3
No. of shells ^2^	0.069	0.094	0.74	0.49	1.3
Infill density ^2^	0.045	0.094	0.48	0.64	1.3
Layer thickness * No. of shells	−0.081	0.053	−1.53	0.18	1.0
Layer thickness * Infill density	0.068	0.053	1.29	0.25	1.0
No. of shells * Infill density	−0.076	0.053	−1.43	0.21	1.0

A regression equation was developed from the ANOVA analysis. The equation was developed using the coefficient of the terms of the response surface model. ^6^

Tensile Test = 0.6648 + 0.0795 A + 0.0540 B + 0.0582 C − 0.0960 AA + 0.0693 BB + 0.0455 CC − 0.0818 AB + 0.0686 AC − 0.0763 BC

Tensile test = 0.6648 + 0.0795 * Thickness of layer + 0.0540 * Number of shells + 0.0582 * Infill density − 0.0960 * Square of layer thickness + 0.0693 * Square of number of shells + 0.0455 * Square of infill density − 0.0818 * Thickness of layer * Number of shells + 0.0686 * Layer thickness * Infill density − 0.0763 * No of shells * Infill density

The response surface model was developed successfully. Using this model, tensile test data for any different combination can be found.

## Conclusions

With DOE of CCD with three input parameters—three levels each of layer thickness, number of shells, and infill density—the test specimens were printed and tensile strength experiments were conducted on specimens. Tensile test data were collected to create a response surface model to successfully predict the tensile strength of 3D printed bronze PLA. Based on the tensile strength data, we can conclude that the stronger specimen has a 0.3 mm of layer thickness, two shells and 40% infill density. The best specimen is that with higher infill density, a higher number of shells and lower layer thickness. In conclusion, infill density played a vital role in the tensile strength of the specimen.

## Author contributions

Chockalingam conceived of the idea, developed the method and performed the verification. Chockalingam encouraged Sugedran to investigate and supervised the findings of this work. All authors discussed the results and contributed to the final manuscript.

## Ethical approval

All procedures used in this project have been approved by Research Ethics Committee (REC) Multimedia University (EA2892021). This work does not involve data collection from human or animal experiments or vulnerable communities.

## Data availability

OSF: Input parameter and their levels, experiment plan, tensile test results, Response Surface Model (RSM) model, ANOVA result and chart.

OSF: Prediction of Tensile Strength of 3D Printed Bronze PLA Part Using Response Surface Modelling.

https://doi.org/10.17605/OSF.IO/NHBDT. ^7^

This project contains the following underlying data:

• Dataset 1: Input parameters, levels, experiment plan. tensile strength results.
• Dataset 2: RSM analysis using Minitab 18 (alternatively R Software), ANOVA results.

Data are available under the terms of the Creative Commons Attribution 4.0 International license (CC-BY 4.0).
