# Supplementary material for: Prediction of Tensile Strength of 3D Printed Bronze PLA Part Using Response Surface Modelling
Source: F1000Res. 2021 Oct 11;10:1030. [Version 1] doi: 10.12688/f1000research.70641.1 (PMC8815010; doi:10.12688/f1000research.70641.1)
Supplement: Peer review history

# Peer review: Reviewer response for version 1

**DOI:** 10.5256/f1000research.74243.r98499

**Published:** 2022-02-03

**Authors:** Raveendran Sundararajan

**Affiliations:** 1 School of Engineering, London South Bank University, London, UK

The finding could lead to the 3D printer sample production for tensile testing for the bronze part only. Layer thickness and infill density were the two main parameters chosen. 

 Wherever possible a graph should be included e.g. Tensile load vs infill thickness and number of layers. Statistical analysis was done in Minitab and this could be more explicit in the report as to how RSM was achieved statistically. 

 This finding could be taken further by using different materials and comparing the data.

Is the work clearly and accurately presented and does it cite the current literature?

Yes

If applicable, is the statistical analysis and its interpretation appropriate?

Partly

Are all the source data underlying the results available to ensure full reproducibility?

No source data required

Is the study design appropriate and is the work technically sound?

Yes

Are the conclusions drawn adequately supported by the results?

Yes

Are sufficient details of methods and analysis provided to allow replication by others?

Yes

Reviewer Expertise:

Thermofluids, pipeflow and sustainable energy

I confirm that I have read this submission and believe that I have an appropriate level of expertise to confirm that it is of an acceptable scientific standard.

# Peer review: Reviewer response for version 1

**DOI:** 10.5256/f1000research.74243.r97370

**Published:** 2021-11-02

**Authors:** Bhagat Singh

**Affiliations:** 1 Jaypee University of Engineering and Technology, Guna, Madhya Pradesh, India

**Overview:** In the present work, authors have tried to investigate the effect of tensile strength on the functionality of the 3D printed parts. They have tried to explore the effects of various process parameters on the tensile strength of these printed parts. In order to achieve this, a response surface model has been developed in terms of the input parameters viz. thickness of layers, number of shells, and infill density. They inferred that infill density and layer thickness are the two prominent parameters governing the tensile strength significantly. The number of shells has the least influence as compared to the other two parameters.

 The manuscript is well written. It can be accepted for indexing in the current form.

Is the work clearly and accurately presented and does it cite the current literature?

Yes

If applicable, is the statistical analysis and its interpretation appropriate?

Yes

Are all the source data underlying the results available to ensure full reproducibility?

Yes

Is the study design appropriate and is the work technically sound?

Yes

Are the conclusions drawn adequately supported by the results?

Yes

Are sufficient details of methods and analysis provided to allow replication by others?

Yes

Reviewer Expertise:

Mechanical Engineering

I confirm that I have read this submission and believe that I have an appropriate level of expertise to confirm that it is of an acceptable scientific standard.
